# Lyso-Gb3 in a Fabry pediatric cohort diagnosed by newborn screening

**DOI:** 10.1016/j.gimo.2026.104366

**Published:** 2026-01-30

**Authors:** Alessandro P. Burlina, Vincenza Gragnaniello, Chiara Edini, Leonardo Salviati, Giulia Polo, Elena Porcù, Natan Meloni, Chiara Cazzorla, Dominique P. Germain, Alberto B. Burlina

**Affiliations:** 1 Neurology Unit, Department of Medicine, St. Bassiano Hospital, Bassano del Grappa, Italy; 2 Division of Inherited Metabolic Diseases, Department of Women's and Children's Health, University of Padua, Padua, Italy; 3 Division of Inherited Metabolic Diseases, Department of Women's and Children's Health, University Hospital of Padua, Padua, Italy; 4 Clinical Genetics Unit, Department of Women's and Children's Health, University of Padua, Padua, Italy; 5 Division of Medical Genetics, University of Versailles and APHP Paris Saclay University, Garches, France

**Keywords:** Fabry disease, Later-onset variants, lyso-Gb3, Neonatal screening, Uncertain significance variants

## Abstract

**Purpose:**

Fabry disease (FD), an X-linked lysosomal storage disorder caused by the deficiency of α-galactosidase A, results in the accumulation of globotriaosylceramide and its deacylated derivative globotriaosylsphingosine (lyso-Gb3). Little is known about lyso-Gb3 levels in pediatric population identified through neonatal screening. This study evaluated levels of lyso-Gb3 at birth and during childhood.

**Methods:**

Plasma lyso-Gb3 levels were monitored annually in 25 males with FD identified by newborn screening. Long-term clinical follow-up was extended up to a maximum of 9 years of age.

**Results:**

A total of 105 plasma samples were collected. For 14 patients with later-onset (LO) variants, 53 lyso-Gb3 values were collected, 50 of which were elevated (mean 2.87 nmol/L, SD 3.46, nv <0.43 nmol/L). We analyzed 52 samples from 11 patients with variants of uncertain significance (VUS), 29 of which were mildly elevated (0.43-1 nmol/L), with overlapping values at birth. Increased lyso-Gb3 levels for LO variants were most pronounced during the first 2 years, after which the values plateaued. Conversely, lyso-Gb3 in VUS increased minimally and then stabilized.

**Conclusion:**

Lyso-Gb3 levels were elevated earlier than clinical symptoms in infants with LO FD, increased significantly in the first 2 years of life, and remained stable during follow-up. Differences in Lyso-Gb3 levels between LO variants and VUS were significant (*P* < .0001).

## Introduction

Fabry disease (FD, OMIM 301500) is a rare X-linked lysosomal storage disorder caused by a deficiency in the enzyme α-galactosidase A (α-Gal A), which is encoded by the *GLA* gene. This deficiency results in the progressive accumulation of globotriaosylceramide and other related glycosphingolipids.^1^^,^^2^

The clinical presentation of FD varies widely according to clinical phenotype, sex, and age. Patients with the classic form of the disease typically present with angiokeratomas, cornea verticillata, neuropathic pain, hypohidrosis, hearing loss, and gastrointestinal symptoms, which can appear early in childhood.^1^, ^2^, ^3^ In adulthood, more severe complications can arise, including significant involvement of the kidneys, heart, and central nervous system (notably, cerebrovascular disease), and the peripheral nervous system. In patients with later-onset (LO)-type FD, cardiac, renal, and neurological symptoms usually appear in adulthood, often leading to delayed diagnosis and treatment.^4^^,^^5^

Lyso-Gb3 has proven useful in confirming diagnosis, assessing disease severity (classic > LO), and monitoring therapeutic efficacy.^6^, ^7^, ^8^ Most studies on lyso-Gb3 have focused on measurements in adult patients with classic FD. However, there is a growing need for more data on lyso-Gb3 levels during infancy, particularly in patients with LO forms diagnosed by newborn screening (NBS).^9^ Despite the increasing implementation of NBS programs for FD, knowledge on the variability of lyso-Gb3 levels at birth, their predictive value for long-term outcomes, and their role in guiding early clinical interventions remains limited.

This study aimed to address this gap by evaluating the levels of lyso-Gb3 and clinical manifestations from the neonatal period to preadolescence in a cohort of male children identified through NBS. We hypothesized that lyso-Gb3 levels in infancy may contribute to early risk stratification, thereby improving personalized disease management.

## Materials and Methods

### Study population

We retrospectively collected data from male children diagnosed with FD through neonatal screening, which has been implemented since 2015 (more than 300,000 newborns).^10^, ^11^, ^12^ The inclusion criteria were as follows: diagnosis made by NBS, reduced α-Gal A activity, identification of a variant in the *GLA* gene, and a follow-up period of at least 1 year. Variants were classified based on published clinical reports and public databases, including ClinVar and Varsome (last access on July 31, 2025)^13^, ^14^, ^15^ Participants were monitored annually using clinical and biochemical evaluations (Table 1). These follow-up assessments were established based on previous recommendations^16^ and modified according to our experience to reduce the risk of over-medicalization in asymptomatic pediatric subjects identified very early through newborn screening.

**Table 1:** Diagnostic workup and follow-up

Diagnostic Confirmation	Baseline Diagnostic Studies	Follow Up Every 12 Months
• Enzyme activity in leukocytes, lymphocytes or plasma • Genetic analysis (patient and parents)	• ECG • Echocardiogram • Renal function tests • Ophthalmologic examination • Plasma lyso-Gb3	• Clinical examination and neurologic evaluation (angiokeratomas, hypohidrosis, gastrointestinal symptoms, limb pain) • Cardiac assessments (ECG, echocardiography, 24-hour Holter ECG) • Kidney (eGFR according to Schwartz formula, microalbuminuria, proteinuria) • Ophthalmologic examination • Plasma lyso-Gb3

### Plasma lyso-Gb3 assay

During outpatient visits, 105 plasma samples were collected, and lyso-Gb3 levels were measured using liquid chromatography-tandem mass spectrometry (LC-MS/MS), as previously described (normal value < 0.43 nmol/L).^17^ All lyso-Gb3 determinations were performed in our laboratory to ensure consistency and comparability of results. The laboratory participates in the ERNDIM (European Research Network for Inherited Metabolic Diseases) quality control program.

### Statistical analysis

Data are presented as mean, standard deviation, and range. The correlations between the variables were evaluated using Student's *t* test. Statistical analyses were performed using GraphPad Prism version 5.00 (GraphPad Software). Statistical significance was set at *P* < .05.

## Results

### Genotype and clinical findings

A total of 33 male newborns with reduced α-Gal A activity were identified through neonatal screening, all of whom carried a variant of the *GLA* gene (HGNC:4296). Of these, 8 were excluded from the study because of a follow-up period of less than one year. Therefore, 25 male infants underwent long-term follow-up.

Molecular testing showed that 14 patients carried genetic variants associated with the LO form (ACMG 4-5), whereas 11 had variants of uncertain significance (VUS)/probably benign variants (ACMG 2-3) (Table 2).

**Table 2:** Characteristics of our cohort of patients: Demographics, genetics, and clinical findings

Patient	Sex	DBS aGalA Activity nmol/l/h	LysoGb-3 at Birth umo/l (nv <0.43)	Genomic Variant	cDNA Variant	Protein Variant	ACMG Score	Interpretation	Age at Last Follow-Up (Years)	LysoGb-3 at Last Follow-Up nmol/l (nv <0.43)	Clinical Manifestations	Therapy
1	M	0.64	1.07	g.14022A>G	c.644A>G	p.Asn215Ser	5	LO	9	4.26	No	No
2	M	0.63	0.82	g.14022A>G	c.644A>G	p.Asn215Ser	5	LO	5	1.97	No	No
3	M	1.4	1.32	g.14022A>G	c.644A>G	p.Asn215Ser	5	LO	5	4.30	No	No
4	M	0.47	2.22	g.14022A>G	c.644A>G	p.Asn215Ser	5	LO	1	4.15	No	No
5	M	0.77	0.3	g.13217G>A	c.640-801G>A^a^		5	LO	8	1.29	No	No
6	M	1.16	0.54	g.13217G>A	c.640-801G>A^a^		5	LO	7	2.1	No	No
7	M	1.2	0.22	g.13217G>A	c.640-801G>A^a^		5	LO	2	0.88	No	No
8	M	0.72	1.02	g.14953G>A	c.1088G>A	p.Arg363His	5	LO	4	1.91	No	No
9	M	0.77	1	g.14953G>A	c.1088G>A	p.Arg363His	5	LO	2	1.49	No	No
10	M	0.75	0.56	g.14953G>A	c.1088G>A	p.Arg363His	5	LO	2	1.71	No	No
11	M	0.73	2.17	g.14931C>T	c.1066C>T	p.Arg356Trp	5	LO	4	3.71	No	No
12	M	1.37	0.85	g.5213G>A	c.153G>A	p.Met51Ile	4	LO	4	0.67	No	No
13	M	1.88	0.44	g.14463A>C	c.868A>C	p.Met290Leu	4	LO	4	0.58	No	No
14	M	0.59	8.38	g.9056T>C	c.272T>C	p.Ile91Thr	5	LO	3	16.6	Cornea verticillata	No
15	M	3.21	0.53	g.11212G>A	c.427G>A	p.Ala143Thr	3	VUS	6	0.54	No	No
16	M	2.76	0.12	g.11212G>A	c.427G>A	p.Ala143Thr	3	VUS	8	0.49	No	No
17	M	2.96	0.31	g.11212G>A	c.427G>A	p.Ala143Thr	3	VUS	8	0.53	No	No
18	M	2.05	0.36	g.11212G>A	c.427G>A	p.Ala143Thr	3	VUS	7	0.56	No	No
19	M	1.51	0.26	g.14932G>A	c.1067G>A	p.Arg356Gln	3	VUS	3	0.43	No	No
20	M	1.63	0.21	g.14932G>A	c.1067G>A	p.Arg356Gln	3	VUS	1	0.56	No	No
21	M	1.28	0.22	g.14451C>G	c.856C>G	p.Leu286Val	3	VUS	6	0.55	No	No
22	M	1.12	0.41	g.14451C>G	c.856C>G	p.Leu286Val	3	VUS	4	0.57	No	No
23	M	3.45	0.27	g.14115C>T	c.737C>T	p.Thr246Ile	3	VUS	8	0.45	No	No
24	M	0.87	0.35	g.9131G>C+ g.11161A>G	c.347G>C+c.376A>G	p.Gly116Ala + p.Ser126Gly	3/2	VUS	5	1	No	No
25	M	1.93	0.47	g.14159G>A	c.781G>A	p.Gly261Ser	3	VUS	2	1.1	No	No

*LO*, later- onset; *VUS*, variant of uncertain significance.

a Also called IVS4+919G>A.

The most prevalent LO variants were c.644A>G (p.Asn215Ser) (*n* = 4 from Europe), c.640-801G>A (g.13217G>A, also called IVS4+919G>A) (*n* = 3 from Asia), and c.1088G>A (p.Arg363His) (*n* = 3 from Africa). Additionally, c.427G>A (p.Ala143Thr) (classified as VUS) was identified in 4 newborns, whereas c.1067G>A (p.Arg356Gln) and c.856C>G (p.Leu286Val) were found in 2 sibling pairs (both classified as VUS). Other variants detected in single individuals included c.272T>C (p.Ile91Thr), c.153G>A (p.Met51Ile), c.1066C>T (p.Arg356Trp), and c.868A>C (p.Met290Leu) (associated with the LO form), as well as c.737C>T (p.Thr246Ile), c.347G>C+c.376A>G (p.Gly116Ala+p.Ser126Gly), and c.781G>A (p.Gly261Ser) among the VUS.

The average follow-up duration for the cohort was 4.8 years (median, 5 years; range, 1-9 years). Specifically, children with LO variants (*n* = 14) had an average follow-up of 4.5 years (median: 4 years, range: 1-9 years), and children with VUS (*n* = 11) had an average follow-up of 5.1 years (median: 6 years, range: 1-8 years).

At the latest follow-up visit, all children were asymptomatic, except for cornea verticillata observed in patient 14 carrying the c.272T>C (p.Ile91Thr) variant, which manifested at the age of 2 years.

None of the participants received any specific treatment for FD.

### Plasma lyso-Gb3

We collected 105 lyso-Gb3 samples over 9 years (September 2015 to December 2024). All patients had at least 2 available plasma samples, with a maximum of 8 samples per patient.

**Figure 1:**
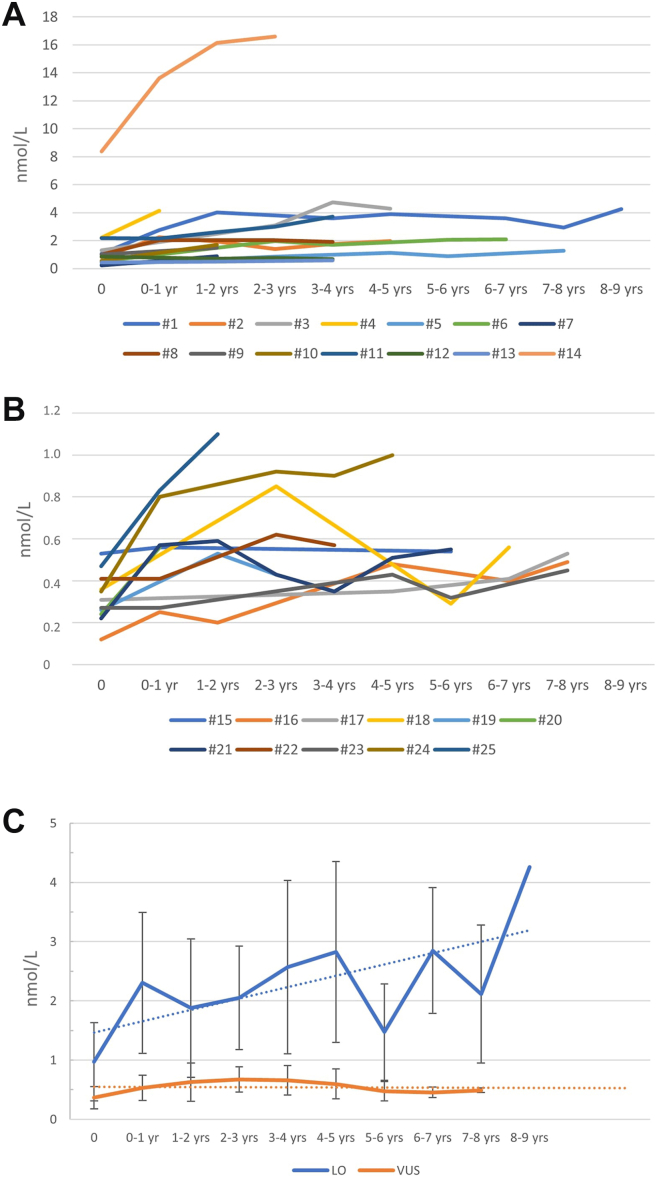
**Plasma Lyso-Gb3 trend in the first decade of life in male individuals carrying later-onset (A) and variants of uncertain significance (B).** Comparison between the 2 groups (mean and SD) (C). Patient 14 was excluded from (C) (see text).

A total of 53 samples were collected from children with LO variants, of which 51 were high (mean 2.87 nmol/L, SD 3.46, range 0.22-16.6 nmol/L). Only 2 nonpathological values were observed at birth (patients 5 and 7, both patients carrying the genetic variant c.640-801G>A) (Figure 1A). Notably, the highest lyso-Gb3 values were observed in patient 14 (8.38 nmol/L at birth, 16.6 nmol/L at 3 years), carrying the c.272T>C (p.Ile91Thr) variant, who was the only individual in our cohort to present early clinical manifestations (cornea verticillata). A family study led to the diagnosis of FD in 2 females (mother and maternal grandmother) and 2 males (maternal uncles). At the age of 31 years, the mother of this individual presented with gastrointestinal symptoms and cornea verticillata. Her plasma lyso-Gb3 level was 3.54 nmol/L. The maternal grandmother, at the age of 61 years, presented with hypertrophic cardiomyopathy, gastrointestinal symptoms, and cornea verticillata, with a plasma lyso-Gb3 level of 2.22 nmol/L. Both the mother and grandmother have started enzyme replacement therapy. The 2 maternal uncles had plasma lyso-Gb3 levels of 11.42 (35 yrs old) and 11.69 nmol/L (37 yrs old), respectively. The first uncle presented with gastrointestinal symptoms, retinal vessel tortuosity on ophthalmoscopic evaluation, and moderate-to-severe high-frequency hearing loss on the left ear (>4 kHz) with normal hearing on the right ear. He started enzyme replacement therapy at the age of 35. The second uncle reported having undergone ablation for an arrhythmic focus. However, he was lost to follow-up because of relocation to another country before a comprehensive evaluation could be performed (see pedigree, Supplemental Figure 1).

For children with VUS, 52 samples were collected, of which only 29 exceeded the cutoff. Notably, all values for individuals with VUS were <1 nmol/L, except for 1 (1.1 nmol/L at 2 years) (Figure 1B).

**Figure 2:**
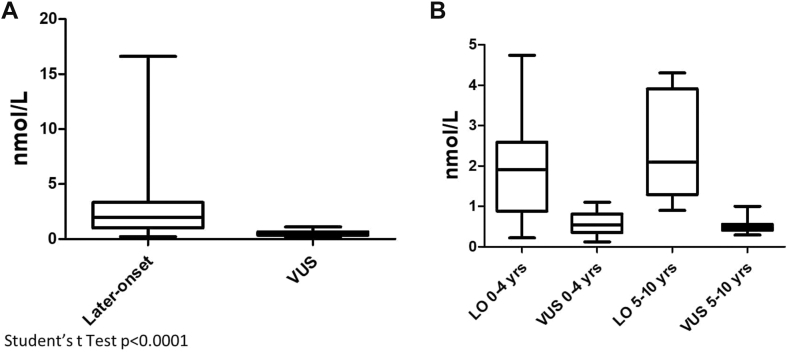
**Box plots of plasma Lyso-Gb3 values in children carrying later-onset and variants of uncertain significance.** All data (A) and according to groups of age (B). A clear distinction between the 2 groups appears at the age of 4 years (B). Patient 14 was excluded from (B) (see text).

At birth, the average values for newborns with LO variants, except c.272T>C (p.Ile91Thr), were 0.97 nmol/L (SD 0.66, range 0.22-2.22), whereas those for newborns with VUS were 0.37 nmol/L (SD 0.19, range 0.12-0.85). Although there was a statistically significant difference between the LO group and VUS group, some values overlapped during the first 4 years (Figure 2A and B).

For LO variants, the mean lyso-Gb3 levels increased from 0.97 nmol/L at birth to 4.26 nmol/L by age 9 years. In contrast, the mean values for VUS remained stable, starting at 0.37 nmol/L at birth and reaching 0.49 nmol/L by age 8.

The greatest increase in values occurred during the first 2 years of life for both LO and VUS patients. After this period, the values remained stable, with variations of less than 0.1 nmol/L per year. Specifically, children with LO variants showed an average increase of 1 nmol/L in the first year and 0.4 nmol/L in the second year, with stable levels in subsequent years. Children with VUS had an average increase of 0.2 nmol/L in the first year, after which their values remained stable (Figure 1C). Figure 1A shows the lyso-Gb3 levels in patient 14 with the c.272T>C (p.Ile91Thr) variant, reported as LO. These values were higher than those of other LO variants and were excluded from the analysis of lyso-Gb3 trends with age because of clinical and biochemical differences compared with those of other individuals with LO variants.

**Figure 3:**
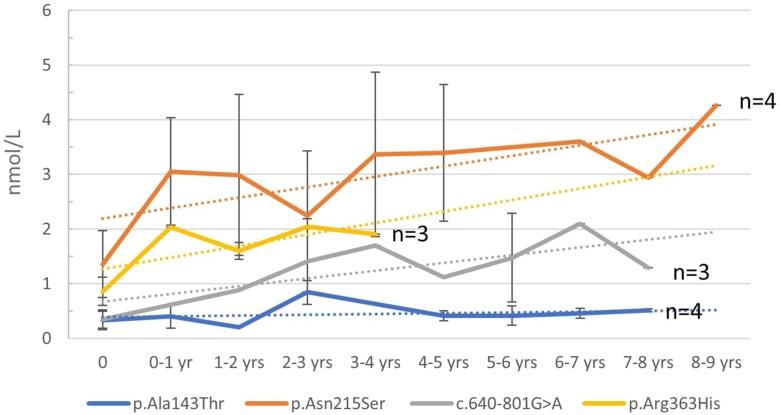
**Plasma Lyso-Gb3 trends in individuals carrying recurrent genetic variants in our cohort of patients**.

We analyzed the lyso-Gb3 levels of the most common variants (3-4 patients) present in our cohort (Figure 3).

Children with the c.427G>A (p.Ala143Thr) variant (*n* = 4) exhibited only a slight increase in lyso-Gb3 levels during the 6 to 8 years of follow-up, with values ranging from 0.12 to 0.56 nmol/L.

In children carrying the c.644A>G (p.Asn215Ser) variant (*n* = 4), the mean lyso-Gb3 value at birth was 1.36 nmol/L (SD 0.6). By the first year, all patients had reached values above 2 nmol/L (mean 3.05, SD 0.99), and 3 of 4 patients had exceeded 4 nmol/L by the age of 4 years.

Among the 3 children with the c.640-801 G>A variant, 2 had normal lyso-Gb3 levels at birth, whereas the third had slightly elevated levels (0.54 nmol/L). The most significant increase occurred within the first 2 years of life, although the values remained below 2 nmol/L.

The 3 children carrying the c.1088G>A (p.Arg363His) variant had birth values ranging from 0.56 up to 1.02 nmol/L, which remained stable throughout the follow-up period.

## Discussion

In this study, we measured plasma lyso-Gb3 levels during the first decade of life in male children with FD who were identified through neonatal screening.

Our results demonstrated that lyso-Gb3 levels at birth were significantly higher in newborns carrying LO variants than in those carrying VUS. Nevertheless, 2 newborns with LO variants presented with normal lyso-Gb3 levels at birth. In fact, lyso-Gb3 levels in the 2 groups may overlap at birth. As previously reported, these findings limit the utility of lyso-Gb3 as a second-tier screening test because normal results do not exclude the possibility of late-onset FD.^11^^,^^18^, ^19^, ^20^

However, during follow-up, all children carrying LO variants showed pathological lyso-Gb3 values starting at the age of 1 year. Moreover, the difference in lyso-Gb3 levels between the LO and VUS groups was evident by tracking trends in the early years of life. Although lyso-Gb3 levels in LO variants increased during the first 2 years and then plateaued, the levels of lyso-Gb3 in the VUS group remained stable below 1 nmol/L, with a plateau reached after 1 year (Figure 1C), resulting in a clear distinction between the 2 groups by the age of 5 years (Figure 2).

Although it is well established in adult patients that plasma lyso-Gb3 concentrations can distinguish male patients with classic and LO forms from those with VUS,^21^, ^22^, ^23^ our study demonstrates that even during the presymptomatic phase in childhood, it is possible to predict the phenotype based on the lyso-Gb3 levels. Indeed, the possibility of measuring lyso-Gb3 levels from birth (by NBS) and assessing clinical follow-up from early life allows for better definition of the phenotype.

In our cohort, patient 14, with the c.272T>C (p.Ile91Thr) variant (classified in international databases as a LO variant), had a higher lyso-Gb3 value at birth (8.38 nmol/L), which further doubled in the first 2 years of life to 16.3 nmol/L, followed by a plateau. Similarly high values were detected in the family members of the patient. At the age of 2 years, the patient manifested cornea verticillata, which affects 94% of patients with the classic form.^24^, ^25^, ^26^, ^27^ Therefore, the onset of this typical ophthalmological sign and elevated levels of lyso-Gb3 challenge the current classification of this genetic variant as LO. This challenging case demonstrates the relevance of NBS in identifying relevant clinical findings.

Our pediatric population presented similar lyso-Gb3 levels to those in a previous extensive Italian study involving 185 male patients with FD (mean age 40.1 years). In this study, the average lyso-Gb3 levels in adult patients were consistent with those in our pediatric cohort, and the authors reported an early plateau. Specifically, the average lyso-Gb3 levels for late-onset variants were 5 nmol/L in adults, compared with 4.26 nmol/L in our cohort; for VUS, 0.8 nmol/L in adults, compared with 0.49 nmol/L at the last follow-up in our patients. Conversely, the average lyso-Gb3 levels for classic FD were 38 nmol/L in adults compared with 16.6 nmol/L in our early symptomatic patient (patient 21).^22^ A previous study conducted on 2 pediatric patients with classic FD demonstrated that plasma LysoGb3 concentrations had higher levels in the symptomatic child (a boy 11-year-old, lyso-Gb3 94.3 ng/ml [119.9 nmol/L]). In the second-born, LysoGb3 showed lower levels when he was an asymptomatic neonate (19.1 ng/ml, 24.3 nmol/L) and almost doubled during the first 5 months of life (37.4 ng/ml, 47.6 nmol/L), reaching approximately 40% of the concentration observed in the symptomatic period.^28^

The early plateau in lyso-Gb3 levels supports its role in phenotype prediction but does not support an effective role for lyso-Gb3 in monitoring disease progression over time. Therefore, we cannot draw any conclusions regarding the value of lyso-Gb3 and the initiation of specific therapy.

Another consequence of the different trends in lyso-Gb3 levels is that, in the early years of life, lyso-Gb3 levels may help to predict the pathogenicity of VUS. The increasing implementation of NBS has led to an increase in the detection of VUS. In this context, lyso-Gb3 could aid in the diagnostic workup of pediatric patients. It can also support decisions regarding the frequency and content of clinical follow-up for patients with VUS.

Finally, we found that lyso-Gb3 levels and trends were similar in male children harboring the same *GLA* variants (Figure 3). The most frequent variants in our study population were c.644A>G (p.Asn215Ser), c.640-801G>A, c.427G>A (p.Ala143Thr), and c.1088G>A (p.Arg363His).

Children carrying the c.427G>A (p.Ala143Thr) variant (*n* = 4) showed only a slight increase in lyso-Gb3 levels during a follow-up period of 6 to 8 years. The nature of this variant, which is common in Europe, has been debated for a long time and is now considered likely benign.^15^^,^^29^ Our findings support the benign nature of this variant.

Children carrying the c.644A>G (p.Asn215Ser) variant (n = 4) showed a similar trend in lyso-Gb3 levels, reaching a plateau at approximately 4 nmol/L.

The 3 children carrying the c.1088G>A (p.Arg363His) variant presented with lyso-Gb3 values ranging from 1.5 2 nmol/L, which remained stable.

Of the 3 patients carrying the c.640-801G>A variant, 2 had normal lyso-Gb3 levels at birth, whereas the third exhibited slightly elevated values (0.54 nmol/L). The greatest increase in lyso-Gb3 levels occurred during the first 2 years of life; however, the values remained below 2 nmol/L. This variant is prevalent in Taiwan, occurring in approximately 1 in 1600 males.^30^ Liao et al^31^ found that all adult males with the c.640-801G>A variant could be distinguished from normal controls by elevated plasma lyso-Gb3 levels. Notably, among 256 individuals carrying the c.640-801G>A variant, approximately 30% of male newborns had normal lyso-Gb3 levels.

These data show that plasma lyso-Gb3 levels correlate with genotype (LO variants > VUS) and that patients with the same genotype show similar trends in lyso-Gb3 progression over time.

The strengths of our study include the long observational longitudinal period in a genetically confirmed FD pediatric cohort from a single center and comprehensive follow-up data. Additionally, the measurement of lyso-Gb3 has always been performed on plasma, which is the gold standard compared with dried blood spot.^32^ Finally, lyso-Gb3 assays were conducted in a single laboratory using standardized technical methods.

A major limitation of this study was the inability to perform neonatal screening based on the measurement of α-Gal enzyme activity to identify female patients. Despite the high incidence of the disease (1:4121 male newborns),^12^ we did not find any patients with the classic form of the disease.

### Conclusion

This study demonstrates that lyso-Gb3 is a noninvasive biomarker of FD that reaches a plateau during the early years of life. Monitoring lyso-Gb3 aids in stratifying at-risk individuals and may provide guidance for a more individualized management approach, as well as help in the classification of VUS. The detection of a plateau for LO variants adds new information on the role of lyso-Gb3 in aging. Future research should aim to investigate longitudinal data to establish lyso-Gb3 levels during the transition from youth to adulthood, and from adulthood to old age in patients with FD. By addressing these gaps, this study establishes a foundation for a more refined approach to FD classification and individualized patient care.

## Conflict of Interest

The authors declare no conflicts of interest.
