# Supplementary figures and images for: Lyso-Gb3 in a Fabry pediatric cohort diagnosed by newborn screening
Source: Genet Med Open. 2026 Jan 30;4:104366. doi: 10.1016/j.gimo.2026.104366 (PMC13158706; doi:10.1016/j.gimo.2026.104366)

Supplementary figure 1: Genetic pedigree of patient #14

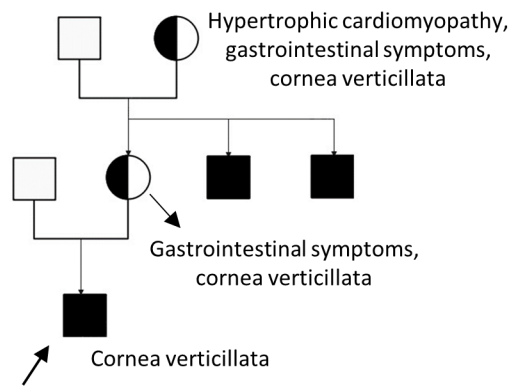

Supplement: Supplementary Figure 1 [file mmc1.pdf]
